# Psychometric Properties and the Network Analysis of the Turkish Version of the Multidimensional Flourishing Scale: Associations with Psychological Distress

**DOI:** 10.3390/bs15060800

**Published:** 2025-06-11

**Authors:** İbrahim Dadandı, Fatih Aydın

**Affiliations:** 1 Department of Educational Sciences, Yozgat Bozok University, Yozgat 66100, Turkey; 2 Department of Educational Sciences, Sivas Cumhuriyet University, Sivas 58140, Turkey; faydin@cumhuriyet.edu.tr

**Keywords:** flourishing, well-being, network analysis, psychological distress

## Abstract

In recent years, the role of flourishing in mental health has gained growing recognition, making the establishment of psychometrically and culturally validated instruments crucial for advancing theory and practice. The aim of this study is twofold: first, to adapt the Multidimensional Flourishing Scale for use in Turkey and investigate its psychometric properties; second, to explore the interrelationships between indicators of flourishing and psychological distress symptoms using network analysis. A total of 529 undergraduate students, comprising 316 females (59.7%) and 213 males (40.3%), with a mean age of 21.65 years (SD = 1.67), participated in the study. The Multidimensional Flourishing Scale, the Flourishing Scale, and the Kessler Distress Scale (K10) were utilized for data collection. Confirmatory factor analysis, Pearson correlation analysis, and the EBICglasso algorithm for network analyses were performed. The findings revealed that the Turkish version of the Multidimensional Flourishing Scale demonstrated a three-dimensional structure consistent with its original version, with satisfactory psychometric properties, including structural and convergent validity as well as reliability. Domain-level network analysis demonstrated that psychological well-being emerged as the most central node within the network, closely followed by psychological distress. Additionally, psychological distress was negatively associated with all domains of flourishing. At the item level, two symptoms of psychological distress, feeling depressed and feeling restless or fidgety, as well as three indicators of flourishing, feeling positive, feeling happy, and perceiving life as full of meaning, emerged as the most central nodes. These findings provide valuable insights into the central features of flourishing and psychological distress, which could potentially guide clinical practice. Further discussion and implications are elaborated upon in the study.

## 1. Introduction

Individuals actively strive to construct a fulfilling life through various means, reflecting a universal desire for satisfaction and well-being. However, the question of what constitutes a “good life” often remains subjective. In the literature, terms such as “happiness,” “mental health,” and “well-being” are frequently used interchangeably to describe a life characterized by positivity (Raibley, 2012). The concept of “flourishing” has garnered attention from researchers over the past quarter-century and emerges as a potential resolution to ongoing debates about well-being. Although it has faced criticism for not being fully developed (Agenor et al., 2017), an expanding body of research provides empirical support for its significance.

Despite the growing interest, a common operationalization for the concept of flourishing has yet to be established. Definitions, drawn from various philosophical frameworks, often include descriptions such as “the experience of life going well” (Huppert & So, 2013), “the state in which all aspects of life are positive” (VanderWeele, 2017), and “the ultimate end in psychology” (Schotanus-Dijkstra et al., 2016). Prominent definitions posit that flourishing is synonymous with positive mental health (Huppert, 2009; Keyes, 2002; Ryff & Singer, 1998). Huppert and So (2013), employing a deductive approach, argue that flourishing is not merely the absence of mental disorders but rather “the exact opposite,” highlighting that the primary component of flourishing is positive functioning in life. Overall, flourishing can be described as an optimal mental state characterized by high levels of both hedonic well-being and eudaimonic well-being (Schotanus-Dijkstra et al., 2016).

Flourishing is one of the central concepts in the positive psychology movement, which has shifted the focus of psychology toward individuals’ happiness, satisfaction, strengths, and sense of meaning in life, moving beyond a sole focus on psychological and psychopathological issues (Seligman, 2011). Over the past two decades, burgeoning scholarly interest in flourishing has resulted in the development of many disparate measures based on diverse perspectives and models (for a review, Novak et al., 2025). Although this heterogeneity in measurement reflects existing theoretical, structural, and conceptual confusion in the field, the diversity of perspectives can stimulate further research, potentially paving the way for a more coherent and unified theory. Additionally, it is essential that measures of flourishing are validated not only psychometrically but also culturally, because while some aspects may be universally applicable, others may hold greater significance within specific cultural contexts (Novak et al., 2025). Therefore, the adaptation of new flourishing measures, such as those developed by Mesurado et al. (2021), to different cultures could significantly contribute to the advancement of theoretical frameworks, while also offering valuable practical benefits.

Another issue is that psychological distress, a mental state characterized by the non-specific symptoms of stress, anxiety and depression (Viertiö et al., 2021), and flourishing are often considered as opposing ends of a mental health continuum and are frequently studied in isolation in academic research (e.g., du Plooy et al., 2019; van Dijk et al., 2017). However, this dichotomy is not absolute; a low level of psychological distress does not necessarily imply a high level of flourishing, or vice versa. In fact, these constructs may function as interrelated subcomponents of overall mental health rather than merely representing opposite poles (Hides et al., 2020). An examination of the intercorrelations among the indicators of psychological distress and flourishing may uncover potential underlying mechanisms linking these constructs and help identify central and bridging indicators that can be targeted in interventions. This issue becomes particularly important in the context of emerging adulthood, a crucial developmental stage marked by emotional, social, and practical challenges such as identity formation, adjustment to university life, financial instability, and the establishment of intimate relationships, which may increase vulnerability to mental health difficulties (Tanner et al., 2009). Research emphasizes that both prevalence and severity of psychological distress symptoms among emerging adults—particularly among university students—are cause for concern (Knapstad et al., 2021; Siddik et al., 2024). Therefore, investigating flourishing in this population, particularly in relation to psychological distress, could offer significant implications for school-based interventions, community-based interventions, and clinical practice. Drawing on these considerations, the present study aims to adapt the Multidimensional Flourishing Scale developed by Mesurado et al. (2021) into Turkish and to investigate the associations between flourishing and psychological distress using network analysis.

## 2. Literature Review

### 2.1. Flourishing: Conceptualization and Assessment Based on Keyes’s Model

Flourishing incorporates both the “eudaimonic” and “hedonic” dimensions of well-being. The eudaimonic perspective of well-being is rooted in Aristotelian ethics, where the Greek term “eudaimonia” signifies the highest human good attained through the pursuit of virtue (Niemiec, 2023). From this viewpoint, human good is seen as an object of desire and choice, with its inherent essence defining its value (Ryan & Deci, 2001). Eudaimonia is regarded as an objective good, distinct from outcomes based on individual experiences or feelings that vary from person to person (Rasmussen, 1999). Thus, this perspective emphasizes both the processes and the substance of living a good life. Conversely, the hedonic perspective of well-being focuses on happiness as a subjective outcome (DeHaan & Ryan, 2014). Within this framework, a good life is characterized by experiences that maximize pleasure for both the mind and body, emphasizing personal desires and self-interests (Ryan & Deci, 2001).

Models conceptualizing flourishing are often grounded in either eudaimonic (Antonovsky, 1993; Jahoda, 1958; Ryan & Deci, 2001; Ryff, 1989) or hedonic perspectives (Bradburn, 1969; Diener et al., 1999; Watson et al., 1988). To develop a more comprehensive and valid understanding of flourishing, it is beneficial to integrate these two perspectives. In a widely accepted model, Keyes (2002) synthesized Diener et al.’s (1999) conceptualization of subjective well-being (the hedonic component) with Ryff’s (1989) six attributes of psychological well-being, while also incorporating a new dimension of social well-being. Psychological well-being is associated with self-acceptance, positive relationships, personal growth, and a sense of purpose and autonomy in meeting personal needs, while social well-being is characterized by social coherence, self-actualization in a social context, social integration, social acceptance, and social contribution. By incorporating this third dimension, Keyes’s (2002) model delineates three core dimensions of well-being: subjective/emotional well-being, psychological well-being, and social well-being. According to Keyes (2002), individuals who fail to flourish are described as languishing; they may lose interest in various aspects of life, perceive their existence as “empty” or “hollow,” and experience feelings of hopelessness and desperation.

One of the primary measurement tools for assessing well-being is the Mental Health Continuum—Short Form (MHC-SF), developed by Keyes (2015). The MHC-SF consists of 14 items organized into three subscales based on Keyes’s flourishing model: the Emotional, Social, and Psychological Well-Being subscales. The literature presents mixed opinions regarding the structural validity of the MHC-SF. Some researchers have suggested that the bifactor model exhibits superior psychometric properties (Söderqvist & Larm, 2023; Żemojtel-Piotrowska et al., 2018), while others have favored a three-factor model (Doré et al., 2017; Keyes et al., 2008). A recent meta-analysis encompassing 48 studies assessing the properties of the MHC-SF revealed support for both the bifactor and tripartite structures of the scale (Iasiello et al., 2022). The MHC-SF has been translated into numerous languages, including Turkish.

Mesurado et al. (2021) developed the Multidimensional Flourishing Scale (MFS), a new assessment tool based on Keyes’s (2002) framework, but with several key differences. Instead of Keyes’s dichotomous (yes/no) approach, the MFS uses a five-point scale to measure the intensity of flourishing. It also distinguishes between positive and negative emotions on a continuum, rather than relying on broad, generalized items. Additionally, Mesurado operationalizes psychological well-being by focusing on meaning in life, personal engagement, and satisfaction, offering a more nuanced perspective than Keyes and further elaborating on social well-being by including aspects such as societal contribution and relationships. Notably, Mesurado conceptualizes emotional well-being as a dynamic mental state assessed over a two-week period, contrasting with Keyes’s more static approach. Overall, Mesurado’s scale aspires to provide a more detailed and multidimensional understanding of flourishing. From a psychometric standpoint, Mesurado et al. (2021) evaluated the performance of the MFS across six countries with Luso-Hispanic roots, demonstrating good structural validity and internal consistency reliability. The authors also established that the MFS exhibits superior convergent validity compared to both the MHC-SF and Diener et al.’s (2010) Flourishing Scale, adapted into Turkish by Telef (2013). Importantly, the authors investigated the cross-cultural stability of the MFS, reporting that the scale possesses good configural, metric, and scalar invariance, positioning it as a candidate for the global assessment of mental health. Consequently, one aim of the present study is to adapt the MFS to Turkish and examine its psychometric properties, anticipating that this translation will facilitate cross-cultural comparisons and enrich flourishing research within a Turkish sample.

### 2.2. Flourishing and Psychological Distress

Keyes (2002) proposed the dual-continua model, suggesting that flourishing and psychological distress constitute two distinct yet interrelated continua of mental health. While flourishing denotes a positive state of well-being, psychological distress refers to a negative mental state often characterized by symptoms of anxiety, depression, and stress (Wang et al., 2022). Despite their connection, these constructs have often been investigated as separate outcomes in mental health research, resulting in two distinct bodies of literature. Moreover, this duality has been further reinforced by empirical findings showing that positive predictors of well-being also negatively predict psychological distress, and vice versa (e.g., du Plooy et al., 2019; Laurene et al., 2024), highlighting their inverse relationship. However, the dual-continua model suggests four mental health profiles that represent intersection points: (1) flourishing without distress symptoms, (2) flourishing with distress symptoms, (3) languishing without distress symptoms, and (4) languishing with distress symptoms (Keyes, 2002, 2005), thereby encouraging the simultaneous investigation of flourishing and psychological distress.

On the other hand, considering flourishing and psychological distress as opposite poles of a continuum could provide a clear distinction at high levels but may render the intersection points along the spectrum ambiguous. More specifically, at moderate levels, the boundaries between flourishing and distress can become blurred, making it unclear how an individual’s mental state should be conceptualized when neither pronounced flourishing nor significant distress is evident. Indeed, Hides et al. (2020) argued that mental distress and well-being are best understood as distinct subcomponents of overall mental health. Similarly, Winefield et al. (2012) concluded that well-being is not simply the opposite of psychological distress, suggesting that researchers should measure both constructs. In this context, exploring the network of flourishing and psychological distress symptoms may enhance understanding of the intersection points of these continua and provide valuable insight into their interrelations.

Network analysis is widely used to determine the most influential mental health symptoms in psychological assessment tools (Briganti et al., 2019; Lecuona et al., 2022; Mullarkey et al., 2019). Several studies have investigated the network structure of flourishing components (Heshmati et al., 2022; Höltge et al., 2023; Hsu et al., 2024) and their interaction with a wide range of mental health indicators, including occupational burnout and job satisfaction, depressive symptoms, loneliness, neuroticism, and more (Jia et al., 2023; Landvreugd et al., 2024; Zhou et al., 2022). A network analysis of flourishing and psychological distress may offer valuable insights not only into the structural validity of an adapted scale but also into broader theoretical frameworks. However, to our knowledge, there is a gap in the literature on the network structure of flourishing and associated mental health indicators in Turkey. The only research on this subject was conducted by Ekşi et al. (2022), who examined the network structure of flourishing, big-five personality traits, and character strengths (caring, inquisitiveness, and self-control). Consequently, the present study aims to explore the associations between psychological distress and the facets of the MFS at the domain and item levels by employing network analysis.

### 2.3. The Current Study

The present study aims to adapt the Multidimensional Flourishing Scale developed by Mesurado et al. (2021) into Turkish and to evaluate its psychometric properties, specifically focusing on structural validity, convergent validity, and internal consistency reliability within a sample of Turkish undergraduates. Additionally, the study seeks to examine the network structure of flourishing and psychological distress, as well as the strength of their associations at both the domain and item levels.

## 3. Methods

### 3.1. Participants and Procedure

This study employed convenience sampling, a nonprobability method that recruits participants from readily accessible groups to conserve time, money, and effort (Büyüköztürk et al., 2014). Although this approach may limit the generalizability of the results (Ary et al., 2014), implementing probability-based sampling methods is often particularly challenging in social science research. Consequently, it has been emphasized that convenience sampling is the most commonly used method in behavioral sciences research and, in some cases, the only feasible way to collect data (Gravetter & Forzano, 2012; Weathington et al., 2010).

A total of 529 university students from two public universities in Turkey were recruited. The sample comprised 316 (59.7%) female and 213 (40.3%) male students. Of the participants, 108 (20.4%) were freshmen, 122 (23.1%) were sophomores, 130 (24.6%) were juniors, and 169 (31.9%) were seniors. The mean age of the entire sample was 21.84 years (SD = 1.82, range = 18–28 years), with a mean age of 21.62 years (SD = 1.67, range = 18–26 years) for female students and 22.17 years (SD = 1.98, range = 18–28 years) for male students. Most participants (81.8%) described their socioeconomic status as middle, whereas 10.9% reported it as low, and 7.3% as high.

Regarding the adequacy of sample size, Kline (2011) suggests that, for effective parameter estimation in SEM, the sample size should be at least ten times the number of parameters that require statistical estimation in the model. The CFA model tested in this study included 27 distinct parameters; therefore, a sample size of 529 cases was considered sufficient for this analysis. On the other hand, because no established a priori power-analysis procedure currently exists for determining sample size in network analysis, no specific sampling target was set for this analysis prior to data collection; however, to address potential limitations regarding sample size, the accuracy of edge weights and centrality measures was assessed, as suggested in the literature (Hevey, 2018). Additionally, according to Kolaczyk and Krivitsky (2015), the effective sample size (n_eff_) for dense models can be calculated using the formula n_v_ × (n_v_ − 1)/2. In the present study, the item-level network model included 22 nodes; therefore, n_eff_ = 22 × 21/2 = 231, indicating that the current study’s sample size is adequate for network analysis.

First, the necessary permission for the adaptation of the MFS was obtained from Belén Mesurado, the corresponding author of the original scale, as well as from the university administrations to conduct data collection. The data were collected via face-to-face surveys in classrooms. Before the survey packet was distributed to potential participants, they were informed about the aims of the study, the anonymity and confidentiality of the data, and their right to withdraw. Additionally, participants were not paid, nor did they receive any benefits for their involvement; participation in the study was entirely voluntary. This study was conducted in accordance with the Declaration of Helsinki and approved by the Social and Human Sciences Ethics Committee of Yozgat Bozok University (approval number: 15/01, 28 June 2024).

### 3.2. Translation of the Multidimensional Flourishing Scale

The translation of the MFS into Turkish was conducted following the guidelines recommended by Beaton et al. (2000). As emphasized in relevant guidelines, it is better to employ several techniques together to ensure the quality of the adaptation process (Cha et al., 2007; Cruchinho et al., 2024; Ford & Scandura, 2023). Therefore, following the guidelines, committee translation, back-translation, and cognitive interviewing methods were used in the present study. Initially, two translators with distinct expertise independently translated the scale items from English into Turkish. They were both bilingual and faculty members at a university, one in the English Language Teaching Department and the other in the Counseling and Guidance Department. A synthesis of the two versions was subsequently performed by the authors to consolidate the strengths of each translation into a unified draft. Next, this synthesized version was back-translated into English by two other faculty members working at the English Language Department who were completely unaware of the original scale. Finally, an expert committee consisting of faculty members at the English Language Teaching, Turkish Language Teaching, and Counseling and Guidance Departments participated in a focus group discussion and reviewed all the translated and back-translated versions from the previous stages. The composition of the committee was three faculty members from the English Language Teaching Department that previously helped in forward translation (n = 1) and back-translation (n = 2) procedures, one faculty member from the Turkish Language Teaching Department, and three faculty members from the Counseling and Guidance Department, including the authors (n = 2) and one other who assisted previously in forward translation. At this meeting, the original and translated items were compared and discussed in terms of vocabulary and grammar, equivalence of expressions and meanings in the two languages, compatibility of the items with the context of the target population, and retaining the original assessment tool’s conception (Zucoloto et al., 2024). A draft version of the Turkish MFS was created, and cognitive interviews were conducted with a group of volunteer senior students at the Counseling and Guidance undergraduate program (a standard classroom, n = 45). Each item of the scale was projected to the board and discussed with students in terms of clarity and understandability. Thanks to the committees’ careful review, no significant changes were suggested by the students. No pilot study or testing was carried out with the group. The committee later gathered for a final discussion and reached a consensus on the final version of the scale.

### 3.3. Instruments

#### 3.3.1. The Multidimensional Flourishing Scale

The MFS was developed by Mesurado et al. (2021) to assess the concept of flourishing from a multidimensional perspective, based on Keyes’s (2005) theoretical framework. It consists of three subscales, including social well-being, psychological well-being, and emotional well-being, and includes a total of 12 items, with four items per subscale. Respondents rate each item on a five-point Likert scale ranging from 1 (strongly disagree) to 5 (strongly agree). The total score ranges from 12 to 60, with higher scores indicating higher levels of flourishing.

In the validation study of the MFS, exploratory factor analysis identified three factors. This three-factor structure was then confirmed through confirmatory factor analysis with χ^2^ = 114.6, df = 51, *p* ≤ 0.001, χ^2^/df = 2.25, AGFI = 0.93, CFI = 0.95, RMSEA = 0.07, RMR = 0.05, and SRMR = 0.05. Regarding convergent validity, moderate relationships were observed between the Multidimensional Flourishing Scale and two well-established measures of flourishing: the Mental Health Continuum—Short Form (Keyes et al., 2008) and the Flourishing Scale (Diener et al., 2010). Additionally, the MFS demonstrated high internal consistency, with a coefficient H of 0.97 and a total omega of 0.96. For the subscales, the H and omega values were 0.89 for social well-being, 0.90 and 0.89 for psychological well-being, and 0.92 and 0.91 for emotional well-being, respectively (Mesurado et al., 2021).

#### 3.3.2. Flourishing Scale

This scale, developed by Diener et al. (2010) and adapted into Turkish by Telef (2013), is a self-report instrument designed to evaluate human flourishing. It is a single-factor scale that consists of 8 items. Example items include “I lead a purposeful and meaningful life” and “I am optimistic about my future.” Respondents rate each item on a 7-point Likert scale ranging from 1 (strongly disagree) to 7 (strongly agree). The total score ranges from 8 to 56, with higher scores indicating the presence of significant psychological resources and strengths. The adaptation study demonstrated that the Flourishing Scale is a valid and reliable measure of flourishing within Turkish culture. The Cronbach’s alpha for the scale was reported as 0.80, and the test–retest reliability was found to be 0.86 (Telef, 2013).

#### 3.3.3. Kessler Distress Scale (K10)

This scale, developed by Kessler et al. (2002) to measure psychological distress symptoms, was adapted into Turkish by Altun et al. (2019). It is a single-factor scale consisting of 10 items. Participants report how frequently they experienced each item in the last 30 days on a five-point Likert scale (1 = none of the time, 2 = a little of the time, 3 = some of the time, 4 = most of the time, 5 = all the time). Sample items include “In the past 4 weeks, how often did you feel tired out for no good reason?” and “In the past 4 weeks, how often did you feel so sad that nothing could cheer you up?”. Total scores can range from 10 to 50, with higher scores indicating greater levels of psychological distress. Adaptation studies revealed that the Turkish version of the scale is a valid and reliable instrument. The Cronbach’s alpha for the K10 was reported as 0.95, and the test–retest reliability was found to be 0.89 (Altun et al., 2019).

### 3.4. Data Analysis

In this study, data analysis was conducted in two distinct phases. The first phase involved evaluating the psychometric properties of the Turkish version of the Multidimensional Flourishing Scale (TMSF). Initially, a confirmatory factor analysis (CFA) was performed to examine the factorial structure of the scale. Model–data fit was assessed based on the following criteria: a chi-square to degrees of freedom ratio < 5, comparative fit index (CFI) > 0.90, Tucker–Lewis index (TLI) > 0.90, root mean square error of approximation (RMSEA) < 0.08, and standardized root mean square residual (SRMR) < 0.05 (Byrne, 2010; Dash & Paul, 2021; Schumacker & Lomax, 2010). Furthermore, the expected cross-validation index (ECVI) and the Akaike information criterion (AIC) were calculated for model comparison, with smaller values of the AIC and ECVI indicating a better model fit (Schermelleh-Engel et al., 2003). Although skewness and kurtosis values for the scale items (Table 1) suggested a normal distribution, univariate normality does not necessarily guarantee multivariate normality, a critical assumption of SEM (Kline, 2011). Mardia’s test, which was conducted to assess multivariate normality, yielded a value greater than 5, indicating that this assumption was not met (Byrne, 2010). Hence, the CFA was performed using the robust maximum likelihood estimation method.

To assess convergent validity, a Pearson correlation analysis was conducted to examine the relationships between scores on the MFS and the Flourishing Scale. In addition, Average Variance Extracted (AVE) and Composite Reliability (CR) were calculated for further evaluation. Next, measurement invariance of the MFS based on gender was tested. Measurement invariance refers to the equivalence in the performance of a psychological assessment tool to capture the given properties across different groups (Schmitt & Kuljanin, 2008). Finally, to evaluate the internal consistency of the TMSF, reliability analysis was performed using Cronbach’s alpha and McDonald’s omega. Descriptive statistics, Pearson correlations, and reliability coefficients were computed using SPSS (version 26), while CFA was conducted using R statistical software (version 4.3.2).

In the second phase of the analysis, a network analysis was conducted to investigate the relationships between indicators of flourishing and psychological distress symptoms. Network analysis is a methodological approach that focuses on the relationships among variables within a system, employing “nodes” to represent psychological variables (e.g., mood states, symptoms, attitudes) and “edges” to indicate the statistical relationships between these variables (Epskamp et al., 2018; Van Borkulo et al., 2014). This approach emphasizes the interactions among different elements, providing a nuanced understanding of complex psychological phenomena that goes beyond traditional latent variable models (Li et al., 2022). It enables researchers and practitioners to detect central features among the variables or measurement items in a network (Gao et al., 2022; Li et al., 2023). These central features might serve as triggers that activate other features in a network, which may give clues to professionals to target specific elements in clinical cases. One of its primary advantages is its ability to facilitate the examination of complex relationships, revealing patterns that traditional methods might miss (Hevey, 2018), while its graphical outputs make findings more accessible to researchers and practitioners (Marcus et al., 2018).

The network’s structure and strength were estimated using the EBICglasso model, which utilizes the Extended Bayesian Information Criterion (EBIC) (J. Chen & Chen, 2008) and the Least Absolute Shrinkage and Selection Operator (LASSO) (Friedman et al., 2008). The tuning parameter was set at 0.5 to achieve a more parsimonious and accurate network configuration. In this network, nodes represented the study variables, and edges depicted the partial correlations between them. Positive correlations were shown with blue lines and negative correlations with red, with thicker edges representing the strength of correlations. Centrality indices, including betweenness (connectivity), closeness (proximity centrality), and strength (influence centrality), were calculated to assess the importance of each node within the network. The stability of node centrality was evaluated using the Correlation Stability Coefficient (CS-coefficient), which is recommended to be above 0.5 for interpretability and stability (Saxena & Banerjee, 2021). Edge stability was assessed using a bootstrapping procedure with 1000 bootstrap samples and 95% confidence intervals, where fewer overlaps indicate greater stability. Network estimation and visualization were conducted using JASP (version 0.18.1.0), while the stability of edge weights and centrality measures was tested using R software (version 4.5.0).

## 4. Results

### 4.1. Psychometric Properties of the Turkish Version of the Multidimensional Flourishing Scale

To investigate the original three-factor structure of the MFS, identified by Mesurado et al. (2021), a CFA was performed using robust maximum likelihood estimation. The initial analysis indicated a poor model–data fit with *X*^2^ = 208.07, *df* = 51, *p* < 0.05; *X*^2^/*df* = 4.08; CFI = 0.92, TLI = 0.89; RMSEA = 0.083, 90CI [0.071, 0.095], *p* > 0.05; SRMR = 0.055, AIC = 15,553.74, and ECVI = 0.56. Following the examination of the modification indices, the model was modified by adding a covariance between the error terms of items 3 and 4. Considering that these two items (see Table S1) both relate to an individual’s motivation to take responsibility and contribute to society, they are likely to share unique variance beyond the common factor. Allowing their residuals to covary may, therefore, improve model fit while preserving the theoretical integrity of the subdimension.

**Figure 1:**
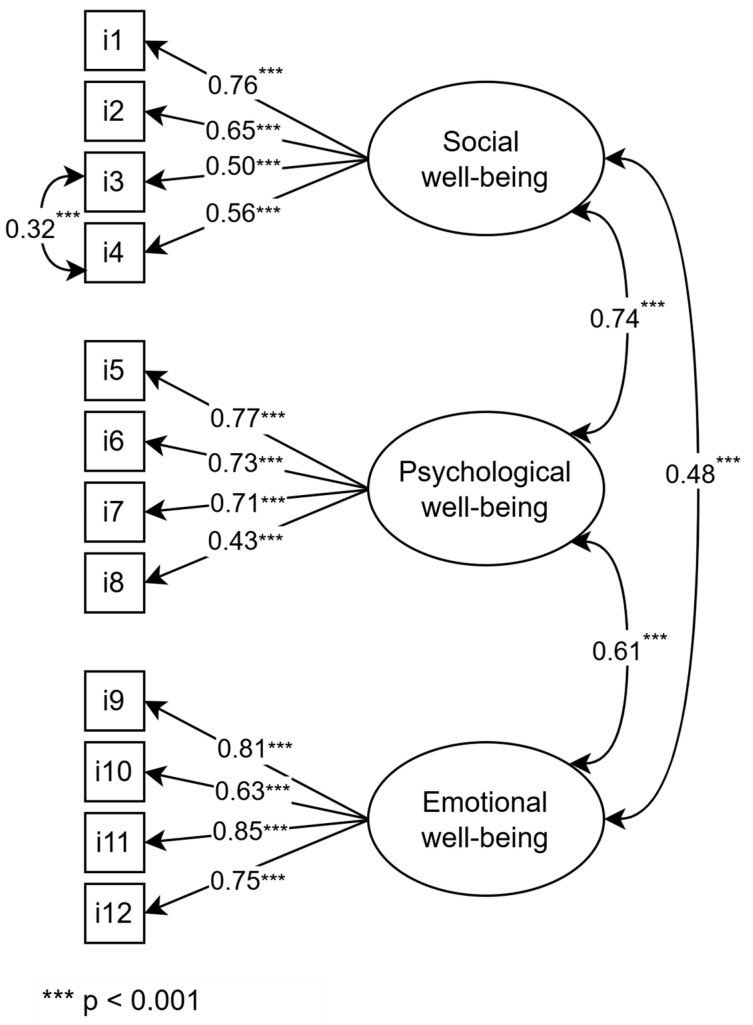
CFA results for the Turkish version of the Multidimensional Flourishing Scale.

The results indicated that the revised model fit the data well: X^2^ = 171.86, *df* = 50, *p* < 0.05; *X*^2^/*df* = 3.43; CFI = 0.94, TLI = 0.92; RMSEA = 0.074, 90CI [0.062, 0.086], *p* > 0.05; SRMR = 0.049, AIC = 15,512.99, ECVI = 0.49. The slightly reduced AIC and ECVI values suggested that the modified model demonstrated a better fit to the data. In this modified model (Figure 1), all standardized factor loadings were statistically significant and exceeded the recommended threshold of 0.40 (Matsunaga, 2010). Additionally, there were significant correlations among the three subdimensions of the MFS. To further evaluate the factor structure of the Turkish version of the MFS, single-factor and bifactor solutions were also considered. CFA results revealed that the single-factor model did not fit the data well (*X*^2^ = 682.53, df = 54, *p* < 0.05; *X*^2^/*df* = 12.64; CFI = 0.70, TLI = 0.63; RMSEA = 0.158, 90CI [0.148, 0.169], *p* > 0.05; SRMR = 0.106, AIC = 16,077.32, ECVI = 1.56). Although the bifactor model yielded better fit indices, its TLI, RMSEA, and SRMR values remained outside acceptable thresholds (*X*^2^ = 180.830, df = 43, *p* < 0.05; *X*^2^/*df* = 4.205; CFI = 0.93, TLI = 0.89; RMSEA = 0.083, 90CI [0.071, 0.096], *p* > 0.05; SRMR = 0.065, AIC = 15,530.412, ECVI = 0.51). These results demonstrated that the three-factor model is superior to the competing single-factor and bifactor models in terms of data fit, indicating that the Turkish version of the MFS retains the same three-factor structure as its original version.

As shown in Table 2, there were moderate positive associations (ranging from 0.46 to 0.70) between the subdimensions of the TMFS and the Turkish version of Diener et al.’s (2010) Flourishing Scale, offering evidence for the convergent validity of the TMFS. Additionally, AVE and CR were calculated to further assess convergent validity. The AVE values for the social, psychological, and emotional well-being subdimensions were 0.39, 0.45, and 0.58, respectively, and the corresponding CR values were 0.72, 0.76, and 0.85. Although CR values for all subdimensions were above the recommended threshold of 0.60, AVE values for the social and psychological subdimensions were below the minimum acceptable threshold of 0.50 (Fornell & Larcker, 1981). However, Fornell and Larcker (1981) also noted that a construct’s convergent validity can still be considered adequate when CR is greater than 0.60, even if AVE is below 0.50. Moreover, further supporting convergent validity, the CFA results indicated a satisfactory model fit and all standardized factor loadings exceeded 0.40 (Cheung et al., 2024). In addition, moderate positive correlations between each TMFS dimension and the Flourishing Scale (Telef, 2013) were observed (Table 2). Taken together, these results indicate that the TMFS demonstrates good convergent validity. The Turkish version of the Multidimensional Flourishing Scale is provided in Table S1.

In CFA, the magnitude of correlations between factors is considered a significant indicator of discriminant validity (Rönkkö & Cho, 2022). In this study, the correlation coefficients among the psychological, social, and emotional well-being subdimensions of the TMFS were all below 0.75, supporting discriminant validity. In addition, the heterotrait–monotrait ratio (HTMT) values, calculated in R, were lower than 0.85 (ranging from 0.46 to 0.74), providing further evidence in support of the discriminant validity (Cheung et al., 2024).

**Table 1:** Factor loadings, descriptive statistics, and item total correlations.

Item No	Mean	SD	Skewness	Kurtosis	Factor Loadings	Item Total Correlations
1	3.68	0.90	−0.82	0.83	0.76	0.60
2	3.38	0.91	−0.66	0.14	0.65	0.61
3	3.39	0.89	−0.60	0.16	0.50	0.50
4	3.64	0.80	−0.74	0.99	0.56	0.51
5	3.59	1.03	−0.81	0.20	0.77	0.69
6	3.51	0.95	−0.63	0.09	0.73	0.66
7	3.12	1.07	−0.30	−0.56	0.71	0.70
8	4.29	0.83	−1.25	1.72	0.43	0.47
9	3.27	1.09	−0.35	−0.51	0.81	0.73
10	3.73	1.08	−0.59	−0.42	0.63	0.60
11	3.17	1.14	−0.29	−0.66	0.85	0.72
12	3.27	1.09	−0.27	−0.45	0.75	0.67

**Table 2:** Descriptive statistics and correlations among the dimensions of the Multidimensional Flourishing Scale and the Flourishing Scale.

Variables	M	SD	1	2	3	4
1. Social well-being	14.09	2.61	1			
2. Psychological well-being	14.48	2.98	0.53 ***	1		
3. Emotional well-being	13.42	3.66	0.36 ***	0.49 ***	1	
4. Flourishing scale	42.06	7.53	0.61 ***	0.70 ***	0.46 ***	1

*** *p* < 0.001.

To evaluate the stability of the TMSF’s factorial structure across gender, a measurement invariance analysis was conducted by comparing scale performance between males and females. Table 3 presents the Satorra–Bentler scaled χ^2^ values and robust fit indices for groups, as well as the results of invariance tests. Results indicated acceptable model fit for males, females, and all nested invariance models. The Satorra–Bentler scaled χ^2^ difference tests (Satorra & Bentler, 2001) showed that the χ^2^ changes were non-significant, indicating that equality constraints across gender did not significantly worsen model fit. Moreover, ΔCFI and ΔTLI values were below the 0.01 threshold, and ΔRMSEA was lower than 0.015 (F. F. Chen, 2007; Cheung & Rensvold, 2002), further supporting measurement invariance across gender. These results suggest that, within the Turkish cultural context, the TMSF demonstrates both metric and scalar invariance across gender, allowing its application across different gender groups.

The reliability of the TMFS was assessed using Cronbach’s alpha and McDonald’s omega coefficients. The Cronbach’s alpha was 0.86 for the overall scale and 0.74, 0.75, and 0.84 for the social well-being, psychological well-being, and emotional well-being subscales, respectively. The McDonald’s omega coefficients were 0.85, 0.74, 0.77, and 0.86 for the social well-being, psychological well-being, and emotional well-being subscales, and the overall scale, respectively. These results suggest that the TMFS had satisfactory internal consistency for both the overall scale and its subscales.

### 4.2. Relationships Between Indicators of Flourishing and Psychological Distress Symptoms

**Figure 2:**
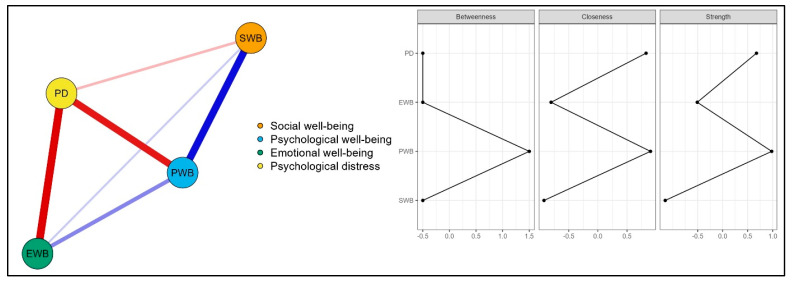
EBICglasso network model and centrality plots for domain-level network analysis.

Two network analyses were conducted to explore the relationships between indicators of flourishing and symptoms of psychological distress at both the domain and item levels. The domain-level network analysis included the total scores for subdimensions of the Multidimensional Flourishing Scale and the Kessler Distress Scale. The EBICglasso network model and corresponding centrality plots for domain-level network analysis are presented in Figure 2. The domain-level network consisted of four nodes and six nonzero edges. The node PD (psychological distress) exhibited the strongest negative edge intensity with EWB (emotional well-being, *r* = −0.39). This was followed by its negative connections with PWB (psychological well-being, *r* = −0.35) and SWB (social well-being, *r* = −0.11). Additionally, there were positive direct associations among the nodes PWB, EWB, and SWB. As shown in Figure 2, PWB emerged as the most central node, displaying the strongest betweenness (1.500), closeness (0.903), and highest strength (0.981) indices. Additionally, the node PD also demonstrated relatively high closeness (0.826) and high strength (0.676) centrality indices (Table S2). The *CS*-coefficient was found to be 0.67, above the recommended threshold of 0.50, which indicates strong stability in node centrality (Kardos et al., 2020). Furthermore, the bootstrapping results demonstrated that the stability of the edge weights and node centralities were relatively high and reliable (Figures S1 and S2).

**Figure 3:**
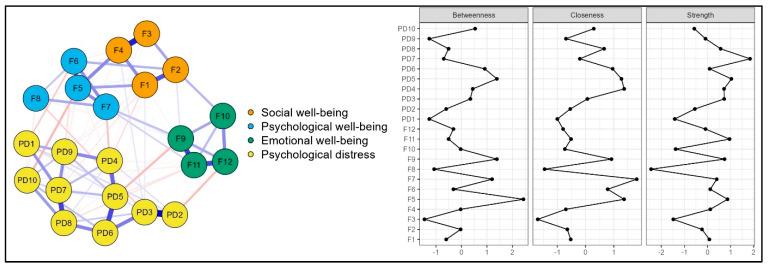
Network model and centrality plots for item-level network analysis.

The item-level network analysis included items from the Multidimensional Flourishing Scale and the Kessler Distress Scale (see Table S3 for detailed specifications of the items and abbreviations used in the network model). The domain-level network consisted of 22 nodes and 98 nonzero edges. As shown in Figure 3, there were positive associations among the nodes related to dimensions of flourishing, as well as between the nodes related to psychological distress. In terms of inverse associations, nodes F9 [“*feeling positive*”] and PD5 [“*In the past four weeks, feeling restless or fidgety*”] had the strongest edge intensity (*r* = −0.13). Similarly, nodes F5 [“*I find my life to be full of meaning*”] and PD10 [“*In the past four weeks, feeling worthless*”], nodes F12 [“*Feeling content*”] and PD2 [“*In the past four weeks, feeling nervous*”], and nodes F7 [“*I am happy with my current lifestyle*”] and PD4 [“*In the past four weeks, feeling hopeless*”] also presented strong edge intensities, with *r* = −0.13, *r* = −0.12, and *r* = −0.11, respectively.

In the item-level network, items PD7 [“*In the past four weeks, feeling depressed*”] and PD5 [“*In the past four weeks, feeling restless or fidgety*”] demonstrated higher levels of strength centrality (1.842 and 1.035, respectively) within the psychological distress domain, while items F11 [“*Feeling happy*”], F5 [“*I find my life to be full of meaning*”], and F9 [“*Feeling positive*”] exhibited relatively higher levels of strength centrality (0.954, 0.867, and 0.736, respectively) in flourishing (Table S4). Furthermore, the bootstrapping results of the network model demonstrated stability in terms of edge weights and centrality measures (Figures S3 and S4).

## 5. Discussion

The present study expanded the knowledge on the psychometric properties of the MFS by following both traditional and novel approaches. Since the MFS is a recently developed measurement instrument, no studies have yet examined its adaptation into different languages. Therefore, current evidence regarding its psychometric properties remains limited. In this context, the present study contributes to the literature by extending the available evidence. The present results supported the three-factor model and provided evidence for the structural validity of the scale. Keyes’s (2002) flourishing conceptualization also built upon three core components of well-being, and the MHC-SF was also recommended to be used as a composition of three factors because it exhibited better structural validity in some studies (Doré et al., 2017; Keyes et al., 2008). The structural validity of the Turkish version of the MFS can be compared with the results obtained from six countries in the original study (Mesurado et al., 2021), where the model fit indexes seem very similar: the CFI was between 0.90 and 0.95, SRMR < 0.05, and *X*^2^*/df* < 5.00. Although Saxena and Banerjee (2021) adapted the MFS for use in India and examined its psychometric properties, their study included only exploratory factor analysis, making direct comparison regarding structural validity infeasible.

Regarding convergent validity, although AVE values for the social and psychological well-being dimensions were lower than the 0.50 threshold, CR values were higher than 0.60, which indicates acceptable convergent validity (Fornell & Larcker, 1981). Moreover, satisfactory SEM fit indices and standardized factor loadings provided further support for convergent validity (Cheung et al., 2024). For additional evaluation, correlations between the emotional, psychological, and social well-being dimensions of the TMFS and the total score on the Turkish version of Diener et al.’s (2010) FS were examined. The results revealed moderate to high correlations (0.46 to 0.70). In the original study, Mesurado et al. (2021) also found moderate correlations between the MFS dimensions and the FS (0.43 to 0.66). In contrast, Saxena and Banerjee (2021) examined convergent validity using the same instruments; however, they reported a correlation between the total score of the MFS (instead of dimension scores) and the FS. Their study revealed a very high correlation, *r* = 0.93. Therefore, the present results are consistent with previous findings and demonstrate that the present study replicated the expected direction and magnitude of the association between the MFS dimensions and the FS.

The present study also revealed that the TMFS exhibits measurement invariance across gender. As extensively reviewed by Putnick and Bornstein (2016), testing measurement invariance in psychological research typically involves three steps: configural, metric, and scalar invariance. Configural invariance refers to whether the same underlying factor structure—specifically, which items load onto which latent constructs—is consistent across different groups. Once configural invariance is established, the next step is to assess metric invariance, which evaluates whether each item loads onto the latent factor to a similar degree across groups. Finally, scalar invariance examines whether differences in item responses between groups reflect true differences in the latent constructs by testing whether item intercepts are equivalent across groups. Accordingly, the Turkish version of the MFS functions similarly for both female and male participants, strengthening evidence for its structural validity. To our knowledge, no prior study has examined the measurement invariance of the TMFS. Thus, the current findings address an important gap in the literature. In contrast, the Flourishing Scale—a competing instrument—has been the subject of several studies examining its measurement invariance across gender, ethno-racial identity, and different populations (Rando et al., 2023; Romano et al., 2020). The present study contributes to the literature on the psychometric properties of the TMFS and reinforces its value among other established instruments.

The internal consistency reliability of the original scale was assessed using the coefficients H and omega. In the current study, both Cronbach’s alpha and McDonald’s omega were calculated. The results indicated good internal consistency reliability, as both α and ω presented high values above 0.70 and 0.80, respectively. The present results are consistent with the original scale and Indian version of the MFS, where α was found to be 0.93 for the whole scale (Saxena & Banerjee, 2021).

The present study also employed a network analysis approach to investigate the associations between flourishing dimensions and psychological distress at the domain and item levels. The findings illustrated that psychological well-being is the most central node at the domain level. It exhibited the highest betweenness, closeness, and strength centrality, indicating that it is the shortest path between other nodes, the closest node to all other nodes in the network, and the average strength of its connections is the highest. Therefore, psychological well-being might represent a core feature of flourishing, and disturbances in positive functioning can trigger a wide range of psychosocial symptoms. Individuals with high psychological well-being tend to perceive life as meaningful, remain committed to daily activities, and cope more effectively with life challenges (Keyes, 2002; Doré et al., 2017; Keyes et al., 2008). Hence, effectively functioning may help individuals regulate adverse emotions. Supporting this, Huppert (2009) emphasized that although external circumstances can harm individuals’ well-being, positive actions and functional attitudes can help them overcome challenges and reduce negative emotions. In this context, positive functioning serves as a central component in sustaining overall well-being. She also noted that while everyone experiences negative emotions such as grief, disappointment, or guilt, persistent negative affect can jeopardize psychological well-being over time. A meta-analysis by Houben et al. (2015) supported this notion, suggesting that low psychological well-being is associated with more inert, unstable, and variable emotional experiences.

Psychological well-being may also promote better social outcomes, enabling individuals to engage more effectively in social environments. In contrast, those who perceive life as meaningless and function ineffectively might struggle with social connection and belonging. Although researchers commonly view psychological well-being as an outcome of supportive social interactions (Gençöz et al., 2004), its potential as a precursor to social engagement is often overlooked. Finally, higher psychological well-being may buffer against psychological distress, making it a promising target for psychological interventions. Accordingly, researchers highlighted that enhancing psychological well-being can help individuals cope with and reduce distress (Azañedo et al., 2020; Di Consiglio et al., 2021).

Following psychological well-being, psychological distress showed the second strongest centrality. While flourishing dimensions had positive edges among them, they demonstrated negative edges with psychological distress, as expected. The strongest positive edge was between psychological and social well-being. This might be because the two constructs together represent eudemonic well-being (Diener et al., 2010; Keyes, 2002). Furthermore, the strongest negative edge was between psychological distress and emotional well-being, followed by psychological well-being. Psychological distress is a maladaptive stress response, comprising negative emotions, heightened physiological reactivity, and compromised functioning (Drapeau et al., 2012). It generally arises from increased threat perception, loss, and overwhelming environmental demands, which impair an individual’s ability to adapt and cope with challenges. This aligns with the strong negative edges between psychological distress and emotional/psychological well-being observed in the present study, which are consistent with the findings from previous literature (Nelson et al., 2010; Winefield et al., 2012).

There is a similar pattern in the item-level network to the domain-level results. Moreover, item-level analysis showed that the indicators of each were associated, which may provide evidence for the existence of latent constructs. At the item level, two items demonstrating high strength centrality, (F9) [“*Feeling positive*”] and (PD5) [“*In the past four weeks, feeling restless or fidgety*”], exhibited one of the strongest negative edges. In essence, positive mood and feelings of nervousness lie at opposite ends of the emotional spectrum. Additionally, these feelings can be easily detected, as people are more attuned to them compared to more complex evaluations of various aspects of their lives. For instance, seeing oneself as a part of a community requires cognitive processing based on prior experiences with others in the environment. Moving on, (F5) [“*I find my life to be full of meaning*”] was another item exhibiting strong centrality within flourishing. This item also demonstrated one of the strongest negative edges with (PD10) [“*In the past four weeks, feeling worthless*”], which is a particularly meaningful finding. According to Baumeister (1991), meaning in life derives from self-worth, efficacy, and purpose. Therefore, the inner sense of being someone who deserves to be loved and belong is a prerequisite for individuals to view life through a more positive lens.

The centrality patterns observed in both domain- and item-level networks offer valuable insights for practical applications. Specifically, the central position of psychological well-being in the network indicates that interventions could be designed to enhance psychological well-being across the broader population, rather than focusing solely on symptom reduction in high-distress groups. Positive psychology-based interventions that target components of psychological well-being, such as meaning in life and purposeful engagement, are likely to produce broader effects across the network, fostering emotional and social well-being while mitigating psychological distress. Furthermore, the item-level network demonstrated that the central indicators of flourishing were “feeling happy,” “I find my life to be full of meaning,” and “feeling positive.” Targeting these core characteristics in interventions and clinical practice may help promote broad improvements in overall psychological health. Similarly, central distress symptoms such as “feeling depressed” and “feeling restless or fidgety” can be strategically targeted in individuals with elevated distress. Reductions in these central symptoms can foster wider symptom relief and accelerate recovery. Finally, these central indicators of flourishing and psychological distress may be used as screening markers to identify at-risk populations and monitor treatment progress in clinical practice.

While the most central features are highlighted here, disregarding the least central attributes may obscure the comprehensive interpretation of the findings. At both the domain and item levels, the total score for social well-being and its corresponding items generally demonstrated noncentral roles and weak edges. Social well-being, as introduced by Keyes (2002) to the conceptualization of flourishing, is considered a vital component of mental health, enabling individuals to thrive throughout life (Carmel, 2019). However, it may not be as central as theoretically assumed. To reach a more valid interpretation, readers may consider extreme cases of languishing or psychiatric conditions such as major depression. Individuals experiencing severe depressive symptoms isolate themselves, withdraw from social engagement, and exhibit a diminished sense of belonging or connection to collective experiences (Osler, 2022). In such circumstances, the core issue lies within the internal psychological processes, suggesting that interventions targeting emotional regulation and meaning-making could be more effective. Nevertheless, it is essential to recognize that overfitting can undermine the performance of the network models. The present study included 529 participants, with 4 nodes in the domain-level network and 22 nodes in the item-level network. These parameters resulted in p/n ratios of 0.008 for the domain-level network and 0.041 for the item-level network, suggesting that overfitting was unlikely to be a significant concern (Williams & Rodriguez, 2022).

### Limitations and Future Directions

The present study provides valuable insights; however, several limitations should be considered when interpreting the findings. First, the study sample consisted exclusively of undergraduate students from two universities, all of whom were young adults. Additionally, most participants were reported to have a middle socio-economic status. Given that experiences and levels of flourishing may vary across different life stages and socio-economic groups, the generalizability of the findings is limited. Future research should aim to include participants from a broader age range—such as adolescents, middle-aged adults, and older individuals—as well as from diverse socio-economic backgrounds, in order to provide a more comprehensive understanding of flourishing. Studies involving more diverse populations may allow for a more generalizable and holistic perspective. Second, the current study did not address cross-cultural validity. As such, future studies may consider incorporating cross-cultural comparisons, particularly by including Turkish participants alongside samples from other cultural backgrounds. Although this study does not directly offer cross-cultural evidence, the data collected from Turkish undergraduates may serve as a foundation for future comparative research.

Another limitation of the study is its use of cross-sectional data, which restricts the ability to draw conclusions about causal relationships. Readers must keep in mind that cross-sectional data do not allow for understanding causal links between factors. Therefore, the current findings should be considered a preliminary investigation. Given their capacity to explore causal relationships, future research could benefit from employing longitudinal or experimental designs to better examine the causal dynamics between flourishing and psychological distress. Additionally, the study relied solely on self-report measures to assess well-being and distress among undergraduate students. Such instruments are susceptible to response biases, including social desirability, which may lead participants to portray themselves in an overly favorable manner. This potential bias should be considered when interpreting the findings. Furthermore, although the present study provides important evidence regarding the psychometric properties of MFS, it does not address its test–retest reliability. Future research should explore this and other psychometric characteristics of the Turkish version of the scale. In addition, the present study provided evidence of the Turkish MFS’s convergent validity; however, the AVE values for the social and psychological dimensions fell below the 0.50 threshold. Although these lower AVE values were compensated by CR values, future studies are encouraged to further evaluate and report convergent validity for these subscales.

Lastly, the study focused exclusively on the network structure of flourishing and psychological distress. Future investigations could expand upon this by incorporating additional indicators of mental health—both positive and negative—such as depression, anxiety, stress, burnout, self-compassion, hope, and mindfulness.

## 6. Conclusions

The present study contributes to the literature by providing evidence that the Turkish version of the Multidimensional Flourishing Scale demonstrates good construct validity, convergent validity, and internal consistency reliability in a sample of Turkish undergraduate students. Furthermore, network analysis revealed that the facets of flourishing-specifically emotional and psychological well-being-exhibited strong negative associations with psychological distress. In contrast, social well-being showed relatively weaker connections with psychological distress at both the domain and item levels, suggesting its less central role in the interplay between flourishing and psychological symptoms within this population.

## Figures and Tables

**Table 3:** Measurement invariance of the TMFS by gender.

Invariance	X^2^	df	ΔX^2^ (df)	CFI	TLI	RMSEA	ΔCFI	ΔTLI	ΔRMSEA
Females	131.447	50		0.933	0.912	0.077			
Males	114.967	50		0.927	0.903	0.083			
Configural	246.440	100		0.930	0.908	0.079			
Metric	252.858	109	7.202(9) ^ns^	0.931	0.917	0.076	0.001	0.009	−0.003
Scalar	263.221	118	8.83 (9) ^ns^	0.931	0.923	0.073	0.000	0.006	−0.003

^ns^: nonsignificant.

## Data Availability

The data supporting this study’s findings are available upon reasonable request from the corresponding author. The data are not publicly available due to ongoing studies.

## Supplementary Materials

The following supporting information can be downloaded at: https://www.mdpi.com/article/10.3390/bs15060800/s1, Table S1. Turkish and English versions of the Multidimensional Flourishing Scale; Table S2. Centrality measures per variable in the domain-level network; Figure S1. Stability of edge weights in the domain-level network; Figure S2. Stability of node centrality in the domain-level network; Table S3. The abbreviation, mean scores, and standard deviations for each variable in the item-level network; Table S4. Centrality measures for each variable in the item-level network; Figure S3. Stability of edge weights in the item-level network; Figure S4. Stability of node centrality in the item-level network.
