# Supplementary material for: Psychometric Properties and the Network Analysis of the Turkish Version of the Multidimensional Flourishing Scale: Associations with Psychological Distress
Source: Behav Sci (Basel). 2025 Jun 11;15(6):800. doi: 10.3390/bs15060800 (PMC12190151; doi:10.3390/bs15060800)
Supplement: Supplementary file 1 [file behavsci-15-00800-s001.zip › behavsci-3666637-supplementary.pdf]

Appendix

Table S1. Turkish and English versions of the Multidimensional Flourishing Scale

	Turkish version	English version
	Yönerge:<br>Aşağıda kendinizle ilgili bir dizi ifade yer almaktadır. Lütfen her ifadeyi dikkatlice okuyun ve ne derecede katıldığınızı 1 ila 5 arasında derecelendiriniz.<br>1. Kesinlikle katılmıyorum<br>2. Katılmıyorum<br>3. Kararsızım<br>4. Katılıyorum<br>5. Kesinlikle katılıyorum	Instructions<br>Below you will find a series of statements about yourself, which you can agree or disagree with. Read them carefully and answer on a scale of 1 to 5, based your experience.<br>1. Strongly disagree<br>2. Disagree<br>3. Neither agree nor disagree<br>4. Agree<br>5. Strongly disagree
	**Sosyal iyi oluş**	**Social well-being**
1.	Kendimi toplumun önemli bir üyesi olarak hissediyorum.	I feel like an important member of society.
2.	Kendimi toplumdaki diğer insanlara yakın hissediyorum.	I feel close to other members of society.
3.	Kendimi toplumsal sorunları çözmeye adarım.	I am committed to addressing the problems faced by society.
4.	Yaptığım işlerin yaşadığım topluma katkı sağladığına inanıyorum.	I believe that my work contributes to the well-being of my society.
	**Psikolojik iyi oluş**	**Psychological well-being**
5.	Anlamlı bir hayatım olduğuna inanıyorum (Bir pusulam, hayatımı tatmin edici kılan ve olası başarısızlıkları ve çelişkileri aşmama yardımcı olan bir anlayışım var).	(I find my life to be full of meaning. [I have a compass, a sense of mission that makes my life fulfilling and helps me to overcome the possible failures or contradictions that I experience]).
6.	Günlük aktivitelerimi (iş, aile aktiviteleri vb.) zevkle yapıyorum.	I am committed to my daily activities (e.g. work and family activities, etc.)
7.	Şu anki hayat tarzımdan memnumum.	I am happy with my current lifestyle.
8.	Ailemle mutluyum.	I am happy with my family.
	**Duygusal iyi oluş**<br>Yönerge:<br>Aşağıdaki iki paralel sütunda son iki hafta içinde nasıl hissettiğini ifade eden bir dizi sıfat göreceksiniz. Her sıfat çifti arasında 5 sayı yer almaktadır. Lütfen hissiyatınızı en yakından tanımlayan sayıyı işaretleyiniz.	**Emotional well-being**<br>Instructions<br>Below you will find a series of adjectives, in two parallel columns, that describe how you have felt over the past two weeks. There are five numbers between each pair of adjectives. Please circle the number that most closely describes your experience.
9.	Negatif 1 2 3 4 5 Pozitif	Negative 1 2 3 4 5 Positive
10.	Aksi 1 2 3 4 5 Arkadaş canlısı	Disagreeable 1 2 3 4 5 Friendly
11.	Üzgün 1 2 3 4 5 Mutlu	Sad 1 2 3 4 5 Happy
12.	Kızgın 1 2 3 4 5 Memnun	Angry 1 2 3 4 5 Content

Note: English version of the items can be found in Mesurado et al. (2021).

References

Mesurado, B., Crespo, R. F., Rodríguez, O., Debeljuh, P., & Carlier, S. I. (2021). The development and initial validation of a multidimensional flourishing scale. *Current Psychology*, *40*, 454-463.https://doi.org/10.1007/s12144-018-9957-9

Table S2. Centrality measures per variable in the domain-level network

Variable	Betweenness	Closeness	Strength
SWB	-0.500	-0.925	-1.149
PWB	1.500	0.903	0.981
EWB	-0.500	-0.804	-0.509
PD	-0.500	0.826	0.676

Note: SWB: Social well-being; PWB: Psychological well-being; EWB: Emotional well-being; PD: Psychological distress

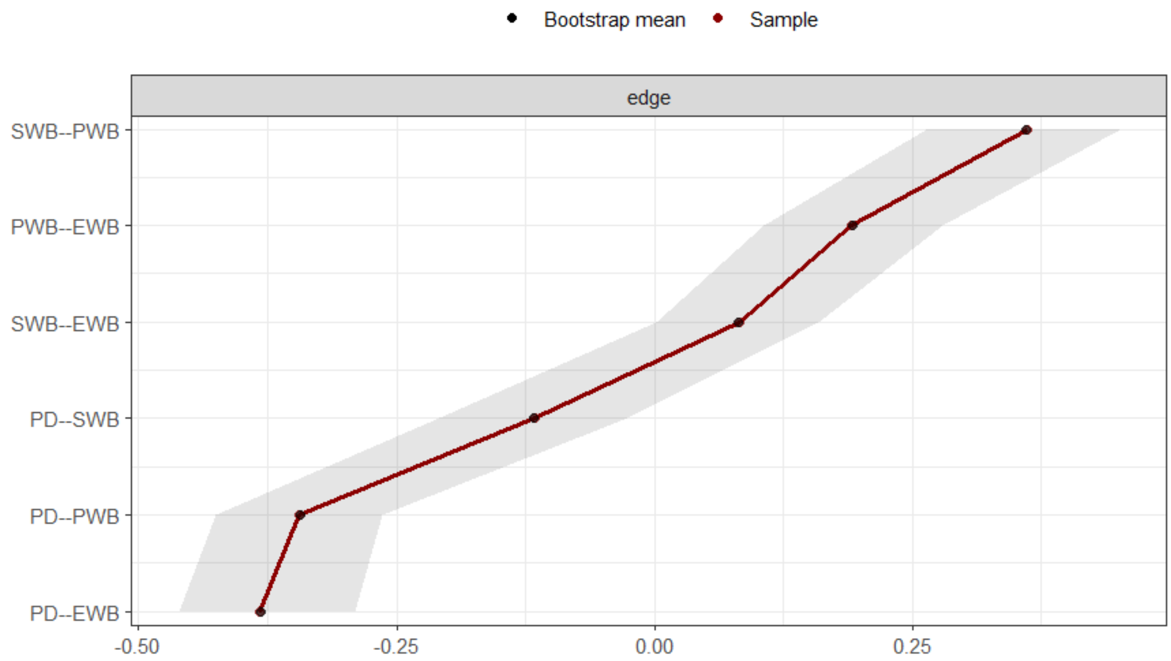

Figure S1. Stability of edge weights in the domain-level network
Note: The red line represents the edge weight values, while the gray area depicts the 95% confidence intervals (CIs).

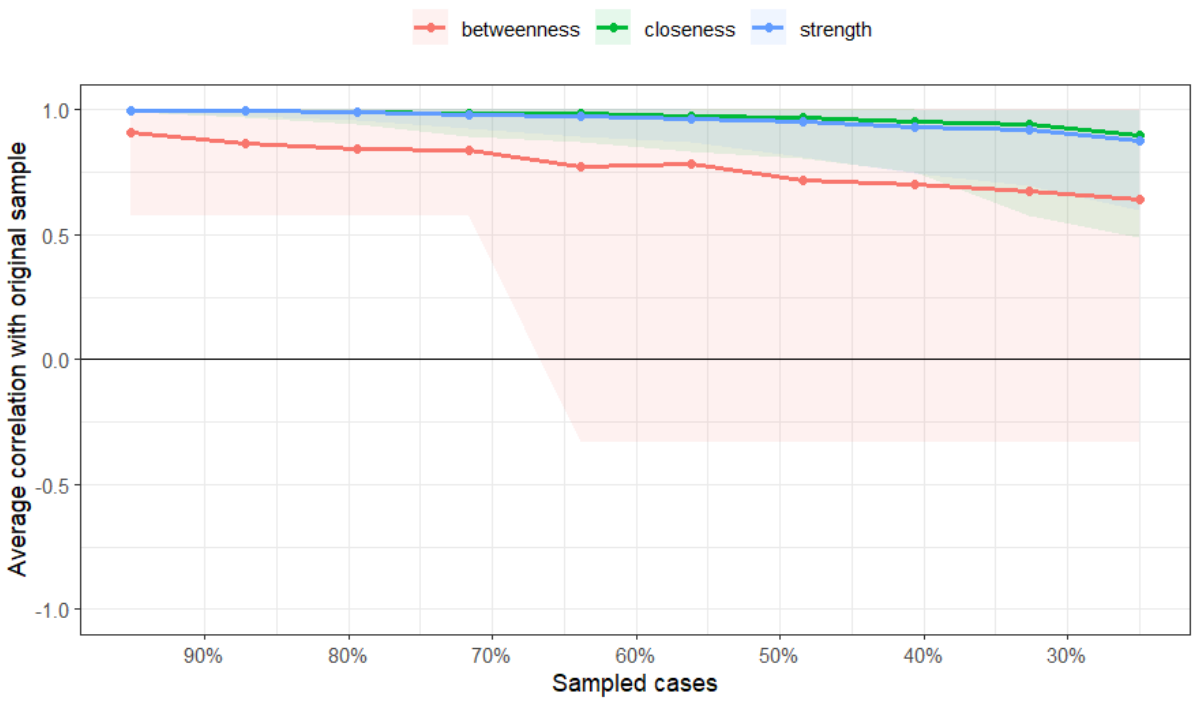

Figure S2. Stability of node centrality in the domain-level network
Note: Average correlation between node strength, closeness, expected influence, and betweenness with sampled cases dropped compared to the original sample. The colored lines show the mean values, and the shaded areas indicate the range from the 2.5th quantile to the 97.5th quantile.

Table S3. The abbreviation, mean scores, and standard deviations for each variable in the item-level network.

Variables	Abbreviation	M	SD
Components of Social Well-Being			
I feel like an important member of society	F1	3.68	.89
I feel close to other members of society	F2	3.38	.91
I am committed to addressing the problems faced by society	F3	3.39	.89
I believe that my work contributes to the well-being my society	F4	3.64	.80
Components of Psychological Well-Being			
I find my life to be full of meaning	F5	3.59	1.03
I am committed to my daily activities (e.g. work and family activities, etc.)	F6	3.51	.95
I am happy with my current lifestyle	F7	3.12	1.07
I am happy with my family	F8	4.29	.83
Components of Emotional Well-Being			
Negative 1 2 3 4 5 Positive	F9	3.27	1.09
Disagreeable 1 2 3 4 5 Friendly	F10	3.73	1.08
Sad 1 2 3 4 5 Happy	F11	3.17	1.14
Angry 1 2 3 4 5 Content	F12	3.27	1.08
Components of Psychological Distress			
In the past 4 weeks, about how often did you feel tired out for no good reason?	PD1	3.49	.97
In the past 4 weeks, about how often did you feel nervous?	PD2	2.96	.85
In the past 4 weeks, about how often did you feel so nervous that nothing could calm you down?	PD3	1.93	.94
In the past 4 weeks, about how often did you feel hopeless?	PD4	3.10	1.06
In the past 4 weeks, about how often did you feel restless or fidgety?	PD5	3.08	.99
In the past 4 weeks, about how often did you feel so restless you could not sit still?	PD6	2.17	1.05
In the past 4 weeks, about how often did you feel depressed?	PD7	2.87	1.17
In the past 4 weeks, about how often did you feel that everything was an effort?	PD8	2.17	1.07
In the past 4 weeks, about how often did you feel so sad that nothing could cheer you up?	PD9	3.13	1.12
In the past 4 weeks, about how often did you feel worthless?	PD10	2.46	1.18

Table S4. Centrality measures for each variable in the item-level network

Variable	Betweenness	Closeness	Strength
F1	-0.598	-0.526	0.081
F2	-0.030	-0.648	-0.238
F3	-1.450	-1.695	-1.481
F4	-0.030	-0.699	0.120
F5	2.431	1.367	0.867
F6	-0.314	0.785	0.118
F7	1.201	1.816	0.401
F8	-1.072	-1.453	-2.443
F9	1.390	0.918	0.736
F10	-0.030	-0.734	-1.380
F11	-0.504	-0.514	0.954
F12	-0.314	-0.797	-0.087
PD1	-1.261	-1.001	-1.418
PD2	-0.598	-0.542	-0.547
PD3	0.349	0.065	0.722
PD4	0.443	1.370	0.717
PD5	1.390	1.274	1.035
PD6	0.917	0.962	0.092
PD7	-0.693	-0.202	1.842
PD8	-0.504	0.658	0.566
PD9	-1.261	-0.695	-0.086
PD10	0.538	0.291	-0.570

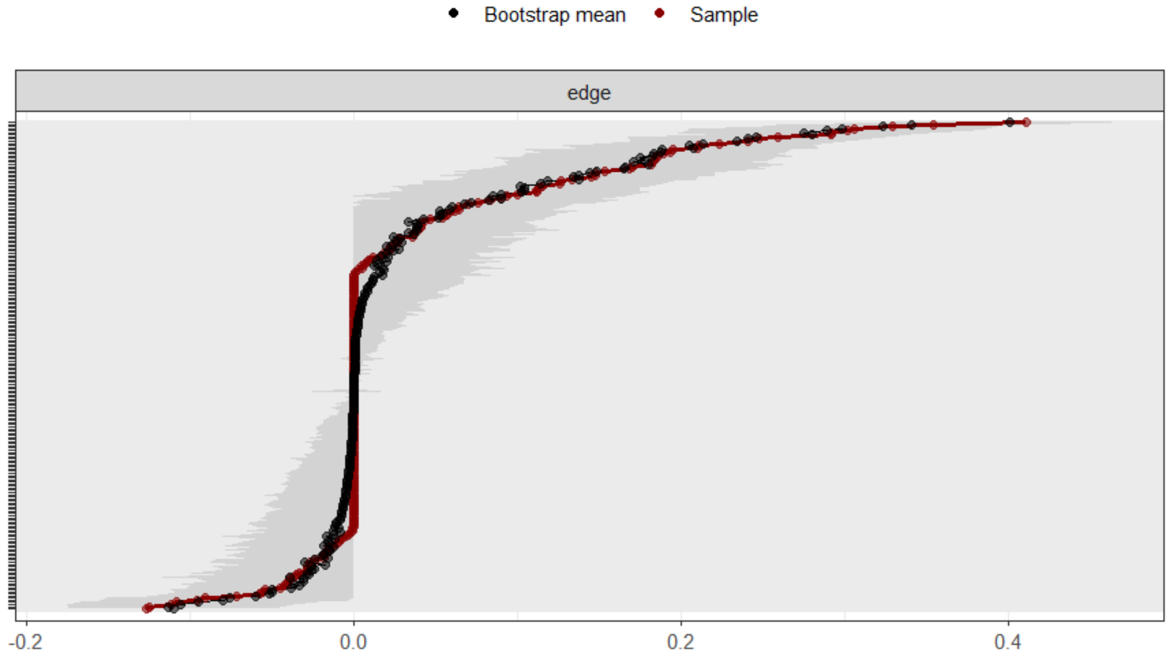

Figure S3. Stability of edge weights in the item-level network
Note: The red line represents the edge weight values, while the gray area depicts the 95% confidence intervals (CIs).

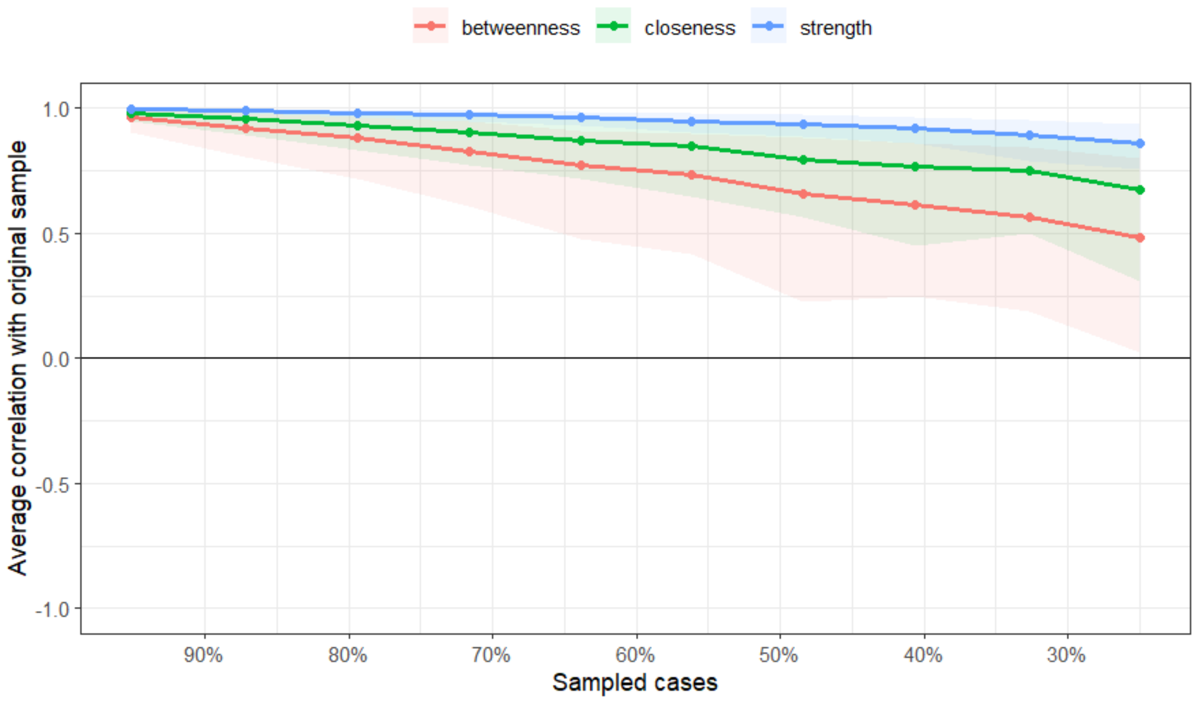

Figure S4. Stability of node centrality in the item-level network
Note: Average correlation between node strength, closeness, expected influence, and betweenness with sampled cases dropped compared to the original sample. The colored lines show the mean values, and the shaded areas indicate the range from the 2.5th quantile to the 97.5th quantile.
